# Microbial assemblages associated with the rhizosphere and endosphere of an herbage, *Leymus chinensis*

**DOI:** 10.1111/1751-7915.13558

**Published:** 2020-03-30

**Authors:** Jin Chen, Daolong Xu, Lumeng Chao, Haijing Liu, Yuying Bao

**Affiliations:** ^1^ Key Laboratory of Forage and Endemic Crop Biotechnology Ministry of Education School of Life Sciences Inner Mongolia University Hohhot 010010 P. R. China; ^2^ State Key Laboratory of Reproductive Regulatory and Breeding of Grassland Livestock Inner Mongolia University Hohhot 010010 P. R. China

## Abstract

Root‐associated microbiomes play significant roles in plant productivity, health and ecological services. However, our current understanding of the microbial assemblages in the rhizosphere and endosphere of herbage is still limited. To gain insights into these microbial assemblages, Illumina MiSeq high‐throughput sequencing was performed to investigate the characteristics of microbial communities of an herbage, *Leymus chinensis*. Hierarchical clustering analysis and principal coordinate analysis (PCoA) results showed that microbial communities of the rhizosphere and endosphere samples were clearly distinguished. Rhizosphere soil communities showed a greater sensitivity than root endosphere communities using linear discriminant analysis (LDA) effect size (LEfSe). Rhizosphere and endosphere communities performed their respective functions in the soil as a cohesive collective, and Rhizobiales were observed to function as generalists. Redundancy analysis (RDA) and variance partitioning analysis (VPA) results revealed that the contribution of the interaction between soil physicochemical parameters and soil enzymes was greater than their individual contributions. In summary, this study is the first to elucidate the microbial diversity and community structure of *L. chinensis* and compare the diversity and composition between rhizospheric and endosphere microbiomes.

## Graphical abstract

In the root‐associated microbiomes of *Leymus chinensis*, the sensitivity of the fungal community was higher than that of the bacterial community, and rhizosphere soil communities showed a greater sensitivity than root endosphere communities. Rhizobiales members were observed to function as generalists in the root‐associated interaction network, and the rhizosphere and endosphere communities performed their respective functions in the soil as a cohesive collective. Moreover, root soil ‐associated microbiomes of *L. chinensis* were significantly affected by the interaction of soil physicochemical parameters and soil enzymes.

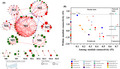

## Introduction

Land plants grow in soil, which exhibits a high abundance and diversity of microorganisms (Thomashow *et al.*, 2018; Chen *et al.*, 2019a). Plants and microorganisms have developed close associations to adapt to various environmental changes and achieve mutual benefit (Lee *et al.*, 2019; Li *et al.*, 2020). In these symbiotic relationships, microorganisms can promote plant growth by providing phytohormones and nutrients, increasing plant tolerance to abiotic stress factors and suppressing plant pathogens (Berendsen *et al.*, 2012; Zhang *et al.*, 2017). In return, plants can provide photosynthetic products from root exudates and tissue debris, including a wide variety of carbon sources, such as secondary metabolites and amino acids (Bais *et al.*, 2004; Philippot *et al.*, 2013). Therefore, exploring the assembly of the root‐associated microbiomes will improve our ability to harness these activities to increase the productivity and quality of cash crops.

Previous studies have used high‐throughput sequencing technology to gain new insights into the microbial composition and organization of different plant microbiomes, including *Arabidopsis*, maize and rice (Bulgarelli *et al.*, 2012; Peiffer *et al.*, 2013; Edwards *et al.*, 2015). Although some studies have reported that the dominant microorganisms in the endosphere (internal plant tissue) are much less diverse than those present in the rhizosphere (the soil region influenced by plant roots) by characterizing the core root microbiome of *Arabidopsis* (Bulgarelli *et al.*, 2012; Lundberg *et al.*, 2012; Peiffer *et al.*, 2013; Schlaeppi *et al.*, 2014), it is unclear whether the root‐associated microbiome of a typical grassland plant, *Leymus chinensis*, follows the same pattern. Naturally, we were curious regarding the differences in the rhizosphere and endosphere microbial community structure, leading us to explore the characteristics of the bacterial and fungal communities of *L. chinensis* in the natural environment.

A previous study demonstrated that the rhizosphere soil located a few millimetres from the roots of plants is profoundly influenced by plant metabolism (Edwards *et al.*, 2015). This phenomenon makes the rhizosphere a ‘hotspot’ environment that results in the corresponding structural and functional differentiation of the rhizospheric microbiome from the external environment (Schlaeppi *et al.*, 2014; Chen *et al.*, 2016). In contrast to the rhizosphere, the endosphere features a highly specific microbiome in which the microbial community structure is relatively stable (Vandenkoornhuyse *et al.*, 2015). Root‐associated microbiomes, including bacterial and fungal communities, build connected and complex ecological networks to respond to changes in the external environment. Additionally, different types of environmental factors may produce the differences in the degree to which root‐associated microbiomes respond to various environmental changes (Shao *et al.*, 2019).

Based on the findings presented above, we hypothesized that (i) the structure and sensitivity of the rhizospheric and endosphere microbial communities of *L. chinensis* may display distinct differences; and (ii) different types of environmental factors may have different degrees of contribution to root‐associated microbiomes. To test these hypotheses, Illumina MiSeq high‐throughput sequencing was performed to investigate the assembly of the root‐associated microbiomes of *L. chinensis* in the Xilin Gol Grassland. The potential core microbiomes, specifically, the biomarkers and generalists of the enriched bacteria and fungi, were also identified. In addition, this is the first study to characterize the rhizosphere and endosphere microbiomes associated with *L. chinensis* and to correlate them with environmental factors. Our results bring new insights into the complexity of root‐associated microbiome communities in grassland herbage.

## Results

### Sequencing results and beta diversity

The 18 composite samples subjected to Illumina MiSeq high‐throughput sequencing assayed in this study generated 3 146 630 raw reads (including 1 613 703 bacterial reads, average of 89 650 per sample; and 1 532 927 fungal reads, average of 85 162 per sample). In this study, 47 683 and 51 051 bacterial and fungal reads per sample (the least number of sequences detected among the assayed samples), respectively, were randomly selected for fair comparisons. The rarefaction curves for the OTUs of all the samples plateaued and are shown in Fig. S1, suggesting that the sequencing depth was sufficient and that all the data were reasonable. The highest richness and diversity were observed in the rhizosphere samples, which exhibited significantly higher taxonomic richness (the *P* values for bacteria and fungi were 0.006 and 0.045 respectively), Chao (the *P* values for bacteria and fungi were 0.037 and 0.002 respectively) and Shannon (the *P* values for bacteria and fungi were 0.021 and 0.036 respectively) compared with that observed in the endosphere samples (Table S1).

**Fig. 1:**
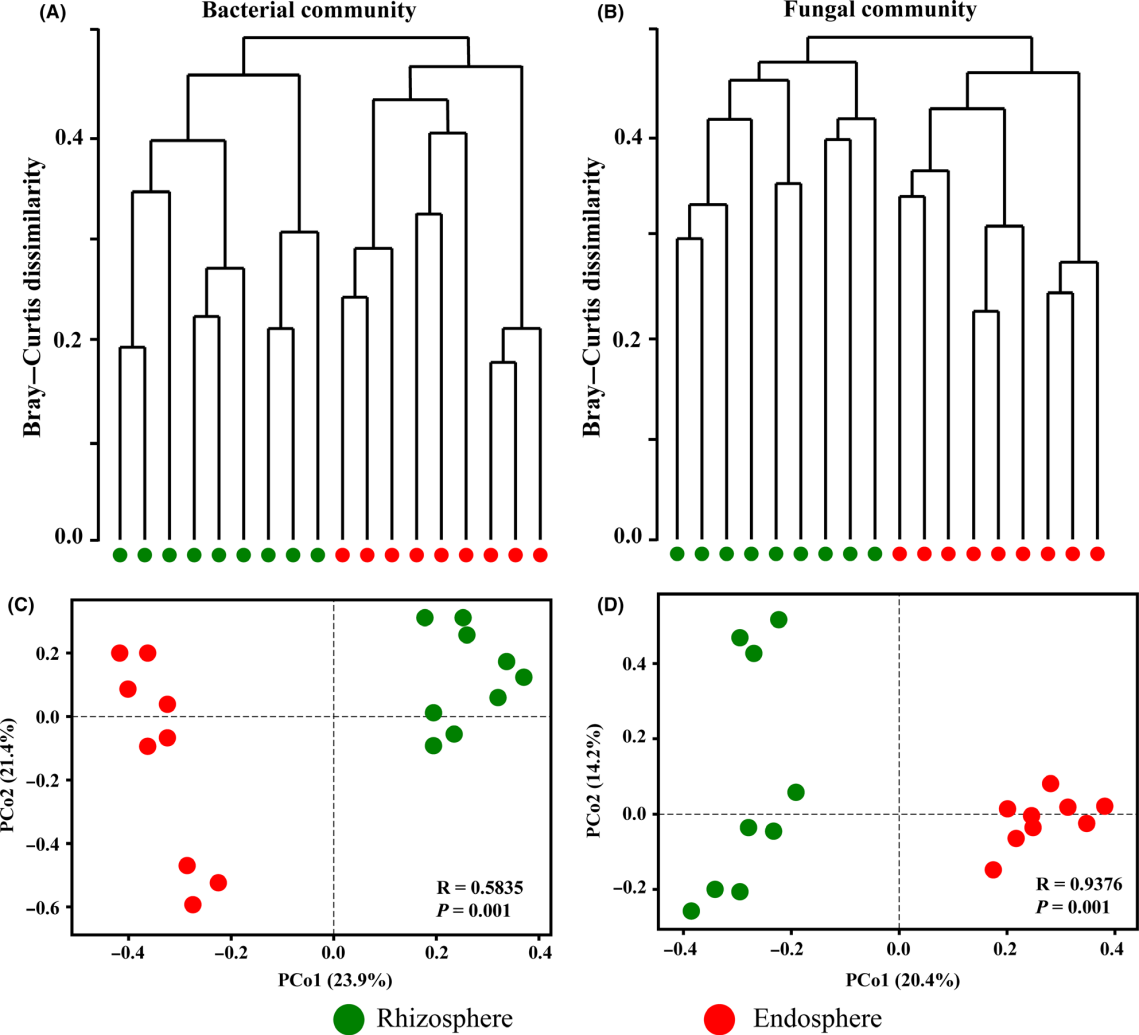
Microbial community differentiation between all samples from the rhizosphere and endosphere. A, B, Hierarchical clustering of bacterial (A) and fungal (B) communities showing the Bray–Curtis dissimilarity of the samples. C, D, Principal coordinate analysis (PCoA) of bacterial (C) and fungal (D) communities based on weighted UniFrac distances between all samples.

We evaluated beta diversity at the OTU level (OTUs defined at a 97% similarity cut‐off). To compare the compositions of identified microbial communities within different plant compartments, hierarchical clustering was performed based on Bray–Curtis dissimilarities at the OTU level. Furthermore, overall similarities in microbial community structures among the samples were displayed using principal coordinate analysis (PCoA; Fig. 1). Hierarchical clustering of the bacterial (Fig. 1A) and fungal (Fig. 1B) communities revealed complete clustering according to plant compartment. The rhizosphere and endosphere samples were clearly distinguished from all the samples according to their respective plant compartment. This pattern was recapitulated by PCoA of weighted UniFrac distances. In this study, PCoA was performed to investigate the separation of the bacterial (Fig. 1C) and fungal (Fig. 1D) communities between all the samples. In both the bacterial and fungal PCoAs, the root‐associated microbiomes between the rhizosphere and endosphere samples separated along the first principal coordinate, and the rhizosphere and endosphere samples revealed a strong clustering of microbial communities.

### Comparison of the microbial community structures and phylogenetic trees of the core microbiomes

In this study, 25 bacterial phyla were identified, where the dominant rhizosphere and endosphere phyla (relative abundance of > 1% in at least in one group) included Proteobacteria (33.62% and 34.41% respectively), Actinobacteria (21.65% and 20.87% respectively), Cyanobacteria (17.43% and 19.32% respectively), Acidobacteria (8.21% and 6.99% respectively), Bacteroidetes (8.22% and 4.17% respectively), Gemmatimonadetes (2.17% and 2.85% respectively) and Chloroflexi (2.76% and 4.51% respectively; Fig. S2a and b). Seven fungal phyla were identified, where the dominant rhizosphere and endosphere phyla included Ascomycota (87.07% and 74.52% respectively), Basidiomycota (5.77% and 16.23% respectively) and Mortierellomycota (3.77% and 0.59% respectively; Fig. S2c and d).

**Fig. 2:**
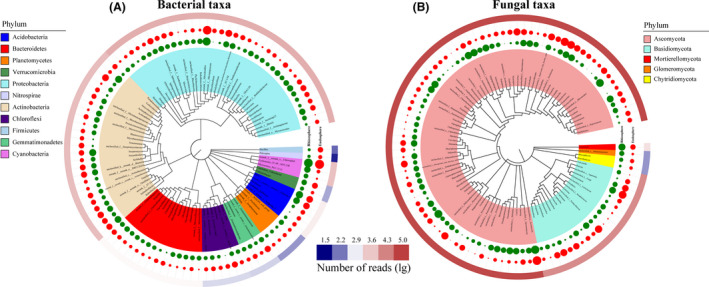
Species abundance of the top 100 genera and phylogenetic relationships of bacterial (A) and fungal (B) taxa between rhizosphere and endosphere samples. The phylogenetic tree is shown at the genus level and coloured at the phylum level. Microbial abundance is indicated in the outer ring with a shape plot (rhizosphere, green circle; endosphere, red circle). The size of the circle represents the sequence log10 reads per genus.

For the microorganisms, we defined the core microbiomes as the 100 most abundant genera among all the samples. Subsequently, a phylogenetic tree was generated using MEGA (neighbour‐joining method, 1000 replicates), and core microbiomes were used to visualize phylogenetic differences with the iToL tool (Fig. 2). The percentages of the total bacterial and fungal communities covered by the core microbiomes were 42.11% and 48.94% in the rhizosphere and 43.99% and 49.04% in the endosphere respectively. Eleven bacterial and 5 fungal phyla were identified as members of the core microbiomes, with only a few branches having bootstrap values below 60, indicating a high reliability and good relationships in the phylogenetic tree. With respect to bacteria, most of the genera in the core microbiomes belonged to the phyla Proteobacteria (35 genera), Actinobacteria (25 genera) and Bacteroidetes (14 genera), with the rest belonging to the phyla Acidobacteria, Gemmatimonadetes, Cyanobacteria, Firmicutes, Planctomycetes, Chloroflexi, Nitrospirae and Verrucomicrobia. With respect to fungi, most of the genera in the core microbiomes were associated with the phyla Ascomycota (77 genera) and Basidiomycota (19 genera), with the rest being belonging to the phyla Chytridiomycota, Mortierellomycota and Glomeromycota.

In addition, we tested the effect of the rhizosphere and endosphere on the normalized sequence counts of members of the core microbiomes using ANOVA. We observed significant plant compartment effects across most of the identified core microbiomes. In the rhizosphere soil of the bacterial community, we observed a significant enrichment (*P* < 0.05) of *Lechevalieria* (3.45%), *Tychonema_CCAP_1459‐11B* (1.96%), *Pseudomonas* (1.05%), *Promicromonospora* (1.12%), *Pseudoxanthomonas* (0.89%) and *Flavobacterium* (0.59%) compared with that observed in the endosphere compartments. In the bacterial root endosphere community, *Bradyrhizobium* (2.83%), *Sphingomonas* (1.68%), *Acidibacter* (1.36%) and *Bacillus* (0.89%) were significantly enriched (*P* < 0.05) compared with that observed in the rhizosphere soil. In the rhizosphere soil of the fungal community, a significant enrichment (*P* < 0.05) in *Darksidea* (9.45%), *Knufia* (3.99%), *Mortierella* (3.66%), *Exophiala* (2.64%), *Humicola* (2.05%), *Pseudogymnoascus* (1.96%) and *Coprinopsis* (1.74%) was observed compared with that detected in the endosphere compartments. In the fungal root endosphere community, a significant enrichment (*P* < 0.05) in *Gibberella* (7.68%), *Chaetomium* (3.90%), *Marasmiellus* (3.64%), *Minimedusa* (1.75%), *Myrmecridium* (1.62%) and *Ceratobasidium* (1.59%) was observed.

### Changes in the relative abundances of biomarkers

**Fig. 3:**
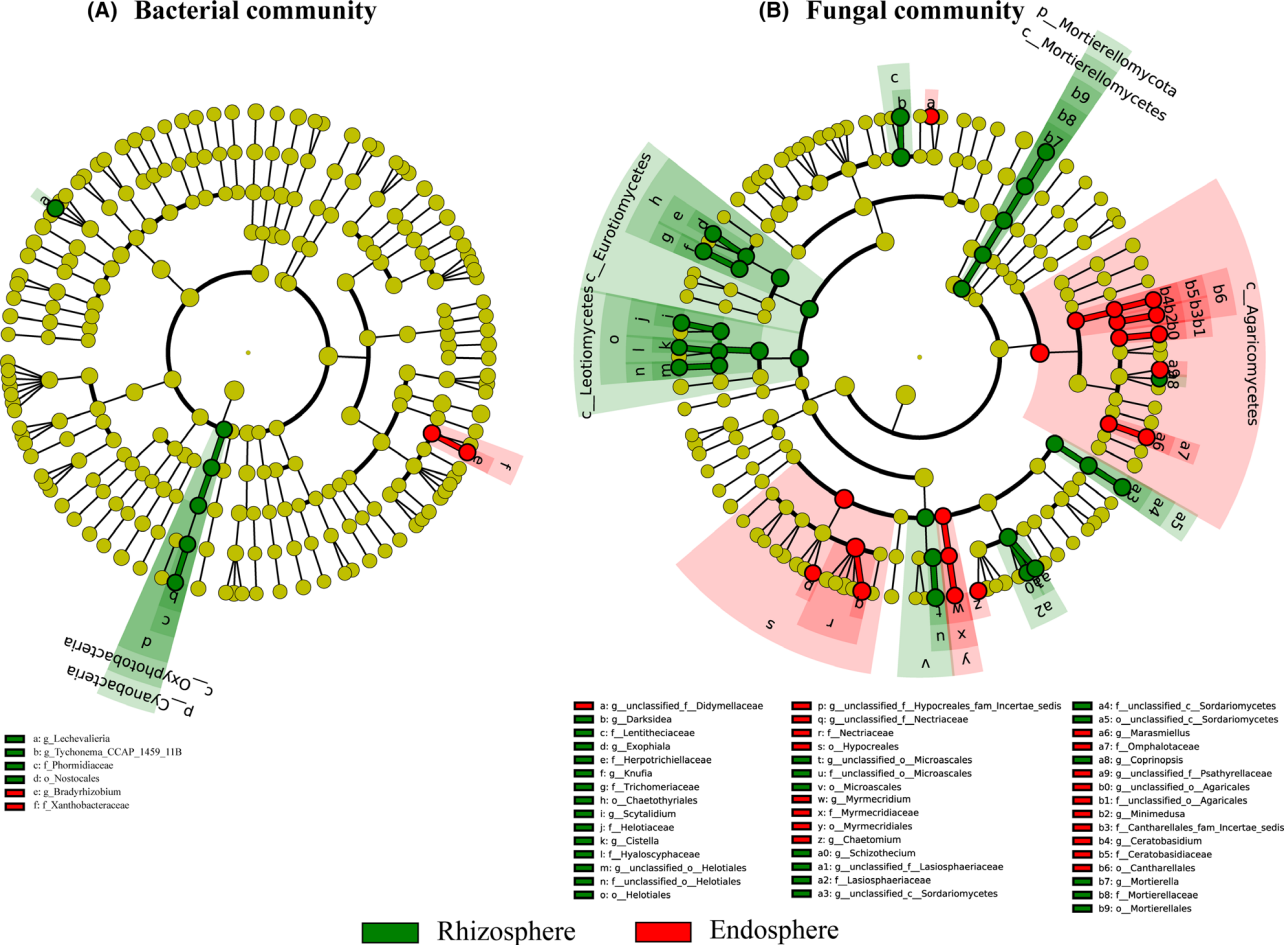
Linear discriminant analysis effect size (LEfSe) of the bacterial (A) and fungal (B) communities with an LDA score higher than 4.0 and *P* values less than 0.05. Cladograms indicate the phylogenetic distribution of microbial lineages associated with the plant compartments. Circles represent phylogenetic levels from kingdom to genus.

The rhizosphere and endosphere communities differed significantly in diversity and richness (*P* < 0.05; Table S1). However, both the rhizosphere and endosphere community compositions were significantly different for the bacterial and fungal communities (Fig. 2). In this study, linear discriminant analysis (LDA) effect size (LEfSe) analysis was performed to identify microbes as biomarkers with LDA scores of > 4 (Fig. 3). These biomarkers showed significant variation in their relative abundances of core genera and were accompanied by considerable changes in response to environmental disturbances. As revealed by LEfSe analysis, when compared with the bacterial community (eight clades, one phyla, one class, one order, two families and three genera), the fungal communities showed a greater sensitivity in the rhizosphere soil environment (51 clades, one phyla, four classes, eight orders, 16 families and 22 genera; Fig. S3).

Further analysis revealed that the rhizosphere communities showed a greater sensitivity to the rhizosphere soil environment than the endosphere communities for both the bacterial and fungal communities, as they exhibited more biomarkers (six biomarkers versus two biomarkers and 31 biomarkers versus 20 biomarkers respectively). Specifically, for the bacterial communities, the proportions of Cyanobacteria, Nostocales, Oxyphotobacteria, Phormidiaceae, *Lechevalieria* and *Tychonema_CCAP_1459_11B* significantly increased in the rhizosphere compartment, while the proportions of Xanthobacteraceae and Bradyrhizobium were significantly enriched in the endosphere compartment. Regarding the fungal community, the proportions of Mortierellomycota, Mortierellomycetes, Leotiomycetes, Eurotiomycetes, *Darksidea* and Lentitheciaceae were significantly enriched in the rhizosphere compartment, while the proportions of Agaricomycetes, Myrmecridiaceae, Ceratobasidiaceae, Cantharellales and Omphalotaceae were significantly increased in the endosphere compartment. The most significant taxa in the endosphere compartment were Nectriaceae and Hypocreales, which had LDA scores of > 5.0.

### Co‐occurrence patterns between the root‐associated microbiomes

**Fig. 4:**
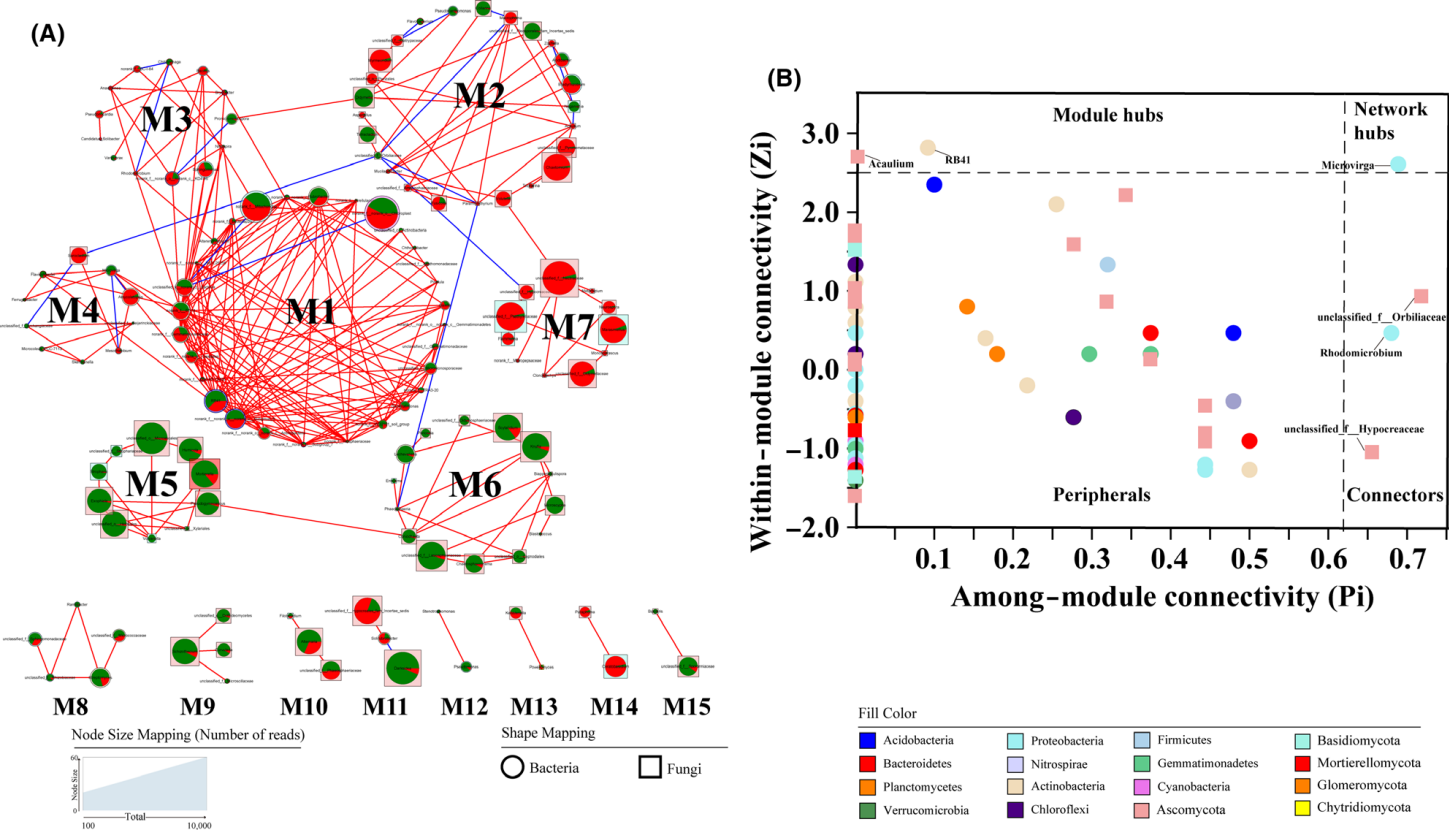
Molecular ecological network analysis revealed the co‐occurrence patterns in the root‐associated microbiomes. A, Genus modules identified relationships based on correlation analysis of core microbiomes. B, Z‐P plots based on topological roles. The links represent strong associations that are significant (***P < 0.001) and strong (Pearson's *r* > 0.8), with the red links indicating positive co‐occurrence relationships, while blue links indicate negative co‐occurrence relationships. The node colours are classified at the phylum level, and the node sizes corresponded to the number of reads. The node shapes represent bacterial (ellipse) and fungal (rectangle) communities, with pie charts are characterized by the proportion of reads in each genus for the rhizosphere (green) and endosphere (red) compartments.

The large number of reads obtained by Illumina MiSeq high‐throughput sequencing technology afforded us the invaluable opportunity to investigate the co‐occurrence patterns within these root‐associated microbiomes using network analysis. The phylogenetic molecular ecological network (pMEN) analysis is a novel RMT‐based framework for studying co‐occurrence patterns that has been used in soil microbial investigations. In the pMEN analysis, the observable association networks between species revealed the correlations of core microbiomes (top 100 bacterial and fungal genera; Fig. 4). A node in pMENs indicates a genus, while a link indicates a co‐occurrence relationship between two connected nodes. The RMT threshold of the molecular ecological network was automatically defined as 0.78, which was higher than the RMT threshold for the majority of networks constructed using this method (Faust and Raes, 2012). As shown in Table S2, networks with 135 nodes were constructed from the root‐associated microbiomes, including 68 nodes for bacteria and 67 nodes for fungi. The average path length (GD), which a value measuring the efficiency of information or mass transport in a network, was 6.391, indicating that the networks constructed in the present study had a small‐world property. The average clustering coefficients (avgCC, a value measuring the extent of a module in a network) of empirical networks (0.291) were significantly higher than the values of corresponding random networks (0.064 ± 0.013), again suggesting the small‐world behaviour of the constructed networks. Furthermore, the modularity value (M, a value that indicates how well a network can be divided into modules) of the empirical networks was 0.649, which was higher than the values of the corresponding randomized networks (0.462 ± 0.009), suggesting that the constructed networks were also modular. All these key topological properties qualified the constructed networks for further analysis.

Two hundred sixty‐five total links of MENs were observed, with an observed positive/negative link ratio (P/N) of 12.95 demonstrating that the root‐associated microbiomes had more positive co‐occurrence relationships. The 200 core microbiomes were clustered into 15 modules (named M1‐15), and each module was considered a functional unit. The abundances of Proteobacteria and Ascomycota were high in most modules (average percentages: 17.04% and 40.74% respectively; Fig. 4A). The two largest modules were M1 and M2, which contained 21.48% (29 nodes) and 18.52% (25 nodes) of the total number of nodes respectively. The bacterial communities were often tightly clustered and formed whole modules, such as M1 and M3. Similarly, the fungal communities were also closely clustered, such as M5, M6 and M7. Nevertheless, some modules were composed of bacterial and fungal communities, such as M2 and M4.

To investigate the potential topological roles of specific nodes within the network, nodes were classified according to their Zi (within‐module connectivity) versus Pi (among‐module connectivity) coefficients (Fig. 4B). Three nodes (accounting for 2.22% of the total nodes in the network) were connectors (connected to multiple modules, Pi ≥ 0.62), while 2 nodes (1.48%) were module hubs (greatly connected to nodes within their modules, Zi ≥ 2.5). Five genera were observed to function as generalists, where *Acaulium* and *RB41* were module hubs and *unclassified_f__Orbiliaceae*, *Rhodomicrobium* and *unclassified_f__Hypocreaceae* were Connectors. Moreover, *Microvirga* members were identified as a network hub (super generalists).

### Relationships between root‐associated microbiomes and environmental factors

**Fig. 5:**
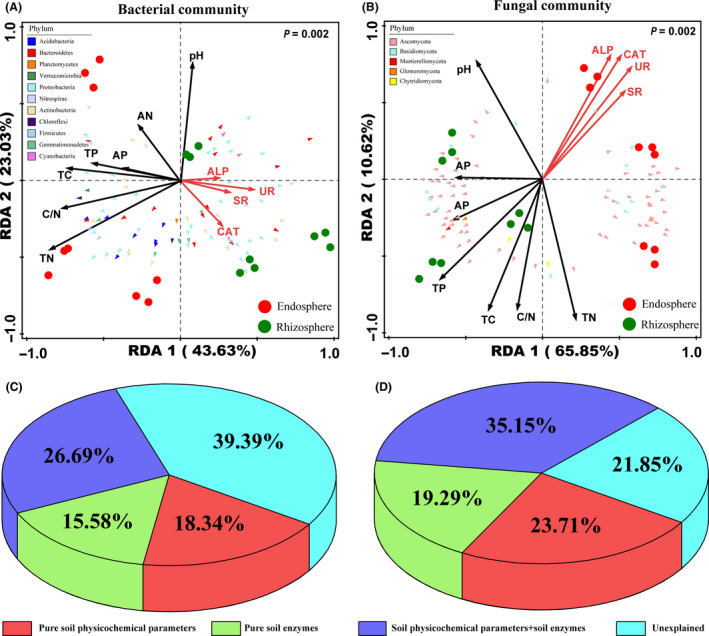
Relationships between root‐associated microbiomes and environmental factors. a, b, Redundancy analysis (RDA) triplot of bacterial (A) and fungal (B) communities; the physicochemical parameters are indicated by black arrows, the soil enzymes are indicated by red arrows, circles with different colours represent different samples, and triangles with different colours correspond to core microbiomes. TC, total carbon; TN, total nitrogen; TP, total phosphorus; C/N, C/N ratio; AP, available phosphorus; AN, ammonia nitrogen; UR, urease; CAT, catalase; SR, sucrase; ALP, alkaline phosphatase. C, D, Variance partitioning analysis (VPA) of bacterial (C) and fungal (D) communities explained by soil physicochemical parameters and soil enzymes.

Redundancy analysis (RDA) was performed to evaluate the taxonomic structure of bacterial and fungal communities and to correlate them with environmental factors (Fig. 5). In general, samples were grouped by the different plant compartments, where the rhizosphere and endosphere samples separated along axis 1. Interestingly, 66.66% of the total variance in the bacterial communities could be explained by the first and second axes (Fig. 5A). Furthermore, the first and second axes together accounted for more than 76% of the total variance in the fungal communities (Fig. 5B). The RDA results clearly showed that all evaluated environmental factors were divided into two groups of explanatory variables and were subsequently named soil physicochemical parameters and soil enzymes. To evaluate the contributions of these two sets of environmental factors, variance partitioning analysis (VPA) was performed to analyse the effects of the above factors on the root‐associated microbiomes. As shown in Fig. 5C and D, the contributions of pure soil physicochemical parameters (including TC, TN, TP, C/N, AP and AN) and pure soil enzymes (including UR, CAT, SR and ALP) to the taxonomic structures of the bacterial and fungal communities were 18.34%–15.58% and 23.71%–19.29% respectively. In contrast, the contribution of the interaction between soil physicochemical parameters and soil enzymes was stronger than that of the individual contributions (48.3% and 46.0% for the bacterial and fungal communities respectively).

## Discussion

*Leymus chinensis* is an ecologically and economically important herbage that is widely distributed in the grasslands of Inner Mongolia and is a native perennial rhizome grass with prominent forage value and great palatability (Zhang *et al.*, 2018). However, knowledge regarding the root microorganisms associated with *L. chinensis* remains limited, although root‐associated microbiomes have been extensively studied in other related plants (Yamamoto *et al.*, 2018; Poudel *et al.*, 2019; Singer *et al.*, 2019). In addition, the microbial community structure of root‐associated microbiomes is different between bacterial and fungal communities, and this difference depends on the chemical and physical properties of the soil. To increase our knowledge regarding root‐associated microbiomes associated with herbage, in this study, we investigated the diversity and community structure of microorganisms present in the rhizosphere and endosphere of *L. chinensis*. The results of this study showed that rhizosphere soil communities showed a greater sensitivity than endosphere communities, with the fungal community being more sensitive than the bacterial community to soil environment. Moreover, core microbiomes in the rhizosphere and root endosphere cooperate to form complex networks of species interactions, which were affected by multiple driving factors, that is the interactions between soil physicochemical parameters and soil enzymes.

Previous reports have demonstrated that the microbial community structure of root‐associated microbiomes was affected by the plant compartment (Edwards *et al.*, 2015; Santos‐Medellin *et al.*, 2017; Yamamoto *et al.*, 2018). Similar results were also obtained for the beta‐diversity analyses in this study. The results of hierarchical clustering and PCoA showed that the rhizosphere and endosphere samples were clearly distinguished from all samples according to their respective plant compartments (Fig. 1). These results were also supported by RDA of the rhizosphere and endosphere samples divided into two different groups (Fig. 5). The OTUs, richness and diversity of bacterial and fungal communities in the rhizosphere from *L. chinensis* were higher than those observed in the root endosphere (Table S1). Root‐associated microbiomes have to overcome the immune defence mechanisms of plants to inhabit the endosphere compartment, which generally leads to reduced density and diversity in the microbial communities compared with that observed in rhizosphere soil communities (Singer *et al.*, 2019). Previous reports have also shown that microbial richness and diversity gradually increase from the root endosphere to the rhizosphere soil and that microbial density is generally lower in the root endosphere than in the rhizosphere (Hacquard *et al.*, 2015; Edwards *et al.*, 2015; Yamamoto *et al.*, 2018).

In all samples, the dominant bacterial phyla were Proteobacteria, Actinobacteria, Cyanobacteria, Acidobacteria, Bacteroidetes, Gemmatimonadetes and Chloroflexi (Fig. 1). The bacterial species belonging to Proteobacteria, Actinobacteria, Bacteroidetes and Acidobacteria could play significant roles in the ecology of *L. chinensis*, as these phyla are also dominant in other herbages (Li and Yang, 2019; Wu *et al.*, 2019; Lu *et al.*, 2019). The dominant fungal phyla were Ascomycota, Basidiomycota and Mortierellomycota. Ascomycota has a high species diversity and fast evolutionary rate and is widely distributed in various habitats (Wang *et al.*, 2010). Yang *et al. *(2019) reported that Basidiomycota could increase plant productivity and soil nutrients by affecting soil microbiome compositions in the alpine and temperate grasslands of China.

Linear discriminant analysis effect size analysis revealed that rhizosphere soil communities were primarily dominated by Cyanobacteria, Nostocales, Oxyphotobacteria, Mortierellomycetes, Leotiomycetes and Eurotiomycetes, which are characteristically isolated from rhizosphere soil samples (Haichar *et al.*, 2008; Berg and Smalla, 2009; Gottel *et al.*, 2011; Bulgarelli *et al.*, 2012). Dominant members of the root endosphere communities included Xanthobacteraceae, Bradyrhizobium, Agaricomycetes, Myrmecridiaceae and Ceratobasidiaceae. All of the above‐mentioned taxa have been isolated from a variety of plant samples, and these species have been demonstrated to have beneficial effects with respect to plant growth metabolism and health (Mark *et al.*, 2006; Delmotte *et al.*, 2009; Fahlgren *et al.*, 2010; Innerebner *et al.*, 2011). The most significant taxa in the root endosphere communities were Nectriaceae and Hypocreales, each having an LDA score of > 5.0 (Fig. 3). The family Nectriaceae (Hypocreales, Sordariomycetes, Pezizomycotina and Ascomycota) includes numerous unique human and plant pathogens, and the majority of these species are facultative or obligate plant pathogens, soil‐borne saprobes or exhibit low virulence, while several members are facultatively fungicolous or insecticolous (Schroers *et al.*, 2011; Graefenhan *et al.*, 2011; Lombard *et al.*, 2015). Several species of the family Nectriaceae have also been reported to be key opportunistic pathogens of animals (Guarro, 2013), while others generate mycotoxins of medical relevance (Rossman, 1996). Mycotoxins produced by these pathogens in the root endosphere of *L. chinensis* indirectly protect the host plant against invasive species and decrease grazing threats by acting as animal pathogens. Furthermore, these soil‐borne saprobes act as primary colonizers in the plant endosphere, having an important role in the functional coupling of grassland ecosystems by decomposing senescent leaves and young litter (Vacher *et al.*, 2016).

Linear discriminant analysis effect size results showed more significant changes in the relative abundances of fungal than bacterial taxa among different samples, indicating that the fungal communities showed a greater sensitivity in the soil environment. In addition, rhizosphere soil communities showed a greater sensitivity than root endosphere communities by harbouring more biomarkers. Compared with fungi, bacteria have a smaller size and a shorter life cycle, making them more susceptible to disturbances from the external environment (Alonso‐Saez *et al.*, 2006; Van der Grinten *et al.*, 2010; Zhang *et al.*, 2020) and leading to their greater sensitivity. The rhizosphere (i.e. the soil close to the root surface) has the closest and most direct interfaces for the exchange of resources between the soil environment and roots (Yu and Hochholdinger, 2018), and is more susceptible to the soil environment than the endosphere (i.e. all inner root tissues).

The phylogenetic molecular ecological network (pMEN) analysis presented that microorganisms had strong syntrophic relationships, indicating that the rhizosphere and endosphere communities performed their respective functions in the soil as a cohesive collective (Fig. 4). In the networks, the number of bacterial and fungal nodes was almost the same (68 versus 67 nodes respectively), suggesting that the bacterial and fungal communities have equal roles in forming a root‐associated interaction network. Proteobacteria and Ascomycota were present in the majority of modules, suggesting that Proteobacteria and Ascomycota play a dominant role in the root‐associated microbiomes of *L. chinensis*. The Z‐P plots showed that *RB41* and Acaulium function as module hubs, and these species have been demonstrated to have important ecological roles in triggering resistance or tolerance to toxicity (Wang *et al.*, 2019). Members of the order Rhizobiales (including the genera *Microvirga* and *Rhodomicrobium*) have been shown to function as generalists, and *Microvirga* species are regarded as super generalists. Rhizobiales (belonging to the phylum Proteobacteria) species are present in root nodules and are involved in the nitrogen‐cycling network (Kuypers *et al.*, 2018). The results of several recent studies have suggested that Rhizobiales also have copiotrophic behaviours towards other microbial communities with respect to soil methane oxidation and phosphate solubilization (Long *et al.*, 2018; Lin *et al.*, 2018; Lin *et al.*, 2019). In summary, it is logical that RB41, Acaulium, Rhodomicrobium and Microvirga function as generalists in the root‐associated microbiomes of *L. chinensis*.

Taking the above information into account, the structure of the rhizosphere and root endosphere communities showed distinct differences. Niche differentiation occurs between the rhizosphere and root endophyte microbiomes in natural ecosystems, which can explain above differences observed in numerous studies (Inceoglu *et al.*, 2010; Lundberg *et al.*, 2012; Bulgarelli *et al.*, 2012; Whitman *et al.*, 2018). In this study, redundancy analysis (RDA) and variance partitioning analysis (VPA) were performed to further assess the niche differences caused by different environmental factors (Fig. 5). A previous study showed that subtle relationships between microbial communities and environmental factors influence microbial community structure and ecosystem‐level processes (Graham *et al.*, 2016). The results showed that the contribution of the interaction between soil physicochemical parameters and soil enzymes was stronger than that of the individual contributions, suggesting that root–soil‐associated microbiomes of *L. chinensis* are affected by multiple driving factors (including soil physicochemical parameters and soil enzymes). Further studies are needed to assess the mechanisms associated with the interaction of these environmental factors in the root‐associated microbiomes of *L. chinensis* to develop its potential for practical application and improve its productivity and quality.

## Conclusions

In the root‐associated microbiomes of *L. chinensis*, the sensitivity of the fungal community was higher than that of the bacterial community, and rhizosphere soil communities showed a greater sensitivity than root endosphere communities. Rhizobiales members were observed to function as generalists in the root‐associated interaction network, and the rhizosphere and endosphere communities performed their respective functions in the soil as a cohesive collective. Moreover, root–soil‐associated microbiomes of *L. chinensis* were significantly affected by the interaction of soil physicochemical parameters and soil enzymes. This information promotes a better understanding of plant–microbe relationships and could be used to further improve the productivity and quality of herbage.

## Experimental procedures

### Study site and sample collection

Sampling was performed at Xilin Gol (42°32′–46°41′N, 111°59′–120°E), Inner Mongolia Autonomous Region, China. Xilin Gol has a continental monsoon climate, with a mean annual temperature of 2°C and a mean annual precipitation of 295 mm (Hao *et al.*, 2019; Chen *et al.*, 2020). The climate of this region is characterized by drought, strong winds and cold temperatures. The terrain of Xilin Gol is low in the north and high in the south, with elevation gently increasing from 900 to 1000 m in the north‐west to 1500 to 2000 m in the south‐east (Li and Yang, 2014). The soil types are primarily chestnut soil, aeolian sandy soil, meadow soil and chernozem. Vegetation primarily includes xeric grasses, such as *Stipa grandis P. Smirn*, *Stipa krylovii Roshev*, *Cleistogenes squarrosa Keng* and *L. chinensis*. *Leymus chinensis*, is naturally and widely distributed in the Xilin Gol Steppe, which has no obvious environmental gradients (Wang *et al.*, 2016).

### Soil sample collection and analysis

In July 2017, 60 healthy individual *L. chinensis* were collected from three random sampling sites across the area (> 10 km apart from each other, 20 plants per sampling site), and rhizosphere soil and root endosphere samples were collected as previously Edwards *et al. *(2015). In brief, roots were collected from *L. chinensis*, and soil was shaken off the roots to leave ~ 1 mm of soil around the roots. Subsequently, the ~ 1 mm of soil was washed off in PBS buffer and maintained as the rhizosphere compartment. The roots were then washed thrice to remove the remaining soil and placed into fresh PBS in a 50‐ml Falcon tube. The clean roots were then sonicated thrice, after which the PBS in the tube was decanted and refilled with fresh PBS to maintain the endosphere compartment. Twenty samples were collected from each sampling site and eventually mixed together to form three composite samples, i.e. 120 samples were mixed into 9 rhizosphere samples and nine corresponding endosphere samples. Then, these rhizosphere and endosphere compartments were retained to extract the microbes. In addition, to investigate the effect of the soil physicochemical parameters and enzymes on microbial communities, we collected bulk soil using a uniform sampling protocol after sieving (< 4 mm). The chemical analysis of the bulk soil is described in the Appendix S1.

### DNA extraction, PCR amplification and Illumina sequencing

Rhizosphere soils and plant roots (0.5 g) were used for DNA extraction using a PowerSoil DNA Isolation Kit (Mo Bio Laboratories, Carlsbad, CA, USA) according to the manufacturer's instructions in triplicate and were subsequently pooled. The quality and quantity of the extracted DNA were assessed using a NanoDrop 1000 spectrophotometer (Thermo Scientific, Wilmington, DE, USA).

To generate bacterial and fungal libraries, universal 16S rRNA gene primers (515F: 5′‐GTGCCAGCMGCCGCGGTAA‐3′ and 806R: 5′‐GGACTACHVGGGTWTCTAAT′) and ITS region primers (ITS1F: 5′‐CTTGGTCATTTAGAGGAAGTAA‐3′ and ITS2R: 5′‐GCTGCGTTCTTCATCGATGC‐3′) were used for bacteria (Parada *et al.*, 2016) and fungi (Adams *et al.*, 2013) respectively. All primers were tagged with an adaptor, a pad and a linker, with a unique barcode sequence used for each sample. The final amplicons were quantified using an AxyPrep DNA Gel Extraction Kit (Axygen Biosciences, Union City, CA, USA) after being detected on 2% (w/v) agarose gels. Equal amounts of purified amplicons were pooled for subsequent sequencing at Allwegene (Beijing, China) using the Illumina MiSeq platform. The Illumina sequences were deposited in the NCBI Sequence Read Archive (accession numbers SRR10807915–SRR10807932 for the bacterial 16S rRNA gene data and SRR10807957–SRR10807974 for the fungal ITS rRNA gene data.).

### Bioinformatics workflow

The sequences obtained from the MiSeq platform were processed using the UPARSE pipeline (Edgar, 2013). The raw sequences were re‐assigned according to their barcodes and were quality‐trimmed using mothur 1.32.2 (Schloss *et al.*, 2009), where chimeric sequences were removed using the UCHIME de novo algorithm. Subsequently, the remaining high‐quality sequences were clustered into operational taxonomic units (OTUs) using the UPARSE algorithm, setting a distance limit of 0.03 (equivalent to 97% similarity) using the open‐reference OTU picking protocol. A representative sequence was aligned using the Python Nearest Alignment Space Termination (PyNAST) against sequences within the SILVA database for bacteria and the Unite database for fungi. The OTUs affiliated with chloroplasts were subsequently removed from the bacterial OTU table, and OTUs that were assigned to non‐fungi, including plant and protozoa, were removed from the fungal OTU table. The sequencing depth for each sample was estimated using rarefaction curves. The effects of sampling on diversity were corrected by rarifying the sequence numbers of each sample to that of the sample with the lowest number of reads (1 613 703 reads for bacteria and 1 532 927 reads for fungi), and three indices, including number of OTUs, Chao and Shannon, were subsequently calculated using mothur (version 1.29.1; www.mothur.org). Based on these data, microbial diversity was estimated using the Shannon index, and microbial richness was estimated using the Chao index.

### Statistical analyses

Statistical analyses in this study were performed using r 3.2.4 (R Core Team, 2016). The data were analysed by one‐way analysis of variance (ANOVA) using spss 25.0 (*P < 0.05, **P < 0.01 and ***P < 0.001 were considered to be significant). All statistical analyses were performed on natural logarithm (log10)‐transformed data to normalize abnormally distributed data. The Bray–Curtis dissimilarity matrix for cluster analysis was analysed using the function ‘vegdist’ in the vegan r package on OTU relative abundances. A phylogenetic tree was generated using MEGA‐X and displayed with the Interactive Tree of Life (itol, http://itol.embl.de). The linear discriminant analysis (LDA) effect size (LEfSe) method (http://huttenhower.sph.harvard.edu/lefse/) was used to detect potential biomarkers at multiple taxonomical levels with an LDA score threshold of > 4.0 and an alpha value for the factorial Kruskal–Wallis test of 0.05. Molecular ecological networks were constructed (Faust and Raes, 2012), where in which the top 100 genera acted as nodes and correlations connecting two genera acted as edges in the network. The role of each genus in the network was characterized by its among‐module connectivity (Pi) and within‐module connectivity (Zi). According to the simplified criteria, all genera were sorted into four subcategories, including network hubs, connectors, module hubs and peripherals. Phylogenetic molecular ecological network (pMENs, http://ieg4.rccc.ou.edu/mena/) analysis was performed to obtain all of the network indexes, and networks were visualized using cytoscape (version 3.7.1). Principal coordinate analysis (PCoA), redundancy analysis (RDA) and variance partitioning analysis (VPA) were performed using canoco 5.0 (Wei *et al.*, 2018; Zhang *et al.*, 2019; Chen *et al.*, 2019b; Wei *et al.*, 2020).

## Conflict of interest

The authors declare no conflict of interest.

## Supporting information


**Appendix S1. **Supplementary material.
