# Supplementary material for: Microbial assemblages associated with the rhizosphere and endosphere of an herbage, Leymus chinensis
Source: Microb Biotechnol. 2020 Mar 30;13(5):1390–402. doi: 10.1111/1751-7915.13558 (PMC7415361; doi:10.1111/1751-7915.13558)
Supplement: Supplementary file 1 — Appendix S1. Supplementary material. [file MBT2-13-1390-s001.docx]

**Supplementary Materials**

**Microbial assemblages associated with the rhizosphere and endosphere of** **an herbage, *Leymus chinensis***

Jin Chen^a,b^, Daolong Xu^a,b^, Lumeng Chao^a,b^, Haijing Liu^a,b^, Yuying Bao^*a,b^

a. Key Laboratory of Forage and Endemic Crop Biotechnology, Ministry of Education, School of Life Sciences, Inner Mongolia University, Hohhot, P. R. China 010010.

b. State Key Laboratory of Reproductive Regulatory and Breeding of Grassland Livestock, Inner Mongolia University, Hohhot, P. R. China 010010.

*Corresponding author Address: Inner Mongolia Univ, 235 West Univ Rd, Hohhot 010021, Inner Mongolia, Peoples R China.

Tel/Fax: +86 471 4492944

E-mail address: [ndbyy@imu.edu.cn](mailto:ndbyy@imu.edu.cn)

Supporting Information Includes:

- 1 Texts
- 2 Table
- 3 Figures

**Captions:**

**Text S1.** Soil sample chemical analysis.

**Table S1.** Sequencing data summary and community diversity.

**Table S2.** Topological properties of the empirical ecological networks (MENs) of root-associated microbiomes and their associate random MENs.

**Fig. S1.** The rarefaction curve analysis of the bacterial (a) and fungal (b) sequences. The curves were constructed using the Shannon index values of the OTUs and the number of reads.

**Fig. S2.** Sunburst showing the hierarchical taxonomic composition of bacteria (a, b) and fungi (c, d). From the inner circle to the outer circle, the phylogenetic levels of kingdom, phylum, class, order, and family are displayed in order. The colors were assigned automatically to distinguish taxa. a, c, The hierarchical taxonomic composition of rhizosphere samples. b, d, The hierarchical taxonomic composition of endosphere samples.

**Fig. S3.** Enriched taxa of the bacterial (a) and fungal (b) communities reaching a linear discriminant analysis (LDA) significance threshold of 4.0.

**Text S1.** Soil sample chemical analysis

The soil moisture content (SM) was determined by heating 10-g composite soil samples at 105  ±  2  °C to a constant weight , then weighting. The soil pH was measured using a pH meter (PHS-3C, Shanghai INESA Instrument Co., Ltd., China) with a 1:5 (w/v) ratio of soil to water following shaking for 1 hour. The soil available nitrogen (AN) and available phosphorus (AP) contents were measured using a UV-Vis spectrophotometer (UV-2450, Shimadzu, Japan). The soil total nitrogen (TN) and total carbon (TC) contents were measured using a CN Analyzer (Vario Max CN, Elementar, Hanau, Germany). The soil total phosphorus (TP), urease (UR), catalase (CAT), sucrase (SR) and alkaline phosphatase (ALP) activities were measured using a soil enzyme kit (Comin Biotechnology Co., Ltd, Suzhou, China) following the manufacturer’s instructions. All measurements of soil samples were performed in triplicate.

**Table S1.** Sequencing data summary and community diversity.

| Sample name | Bacteria | | |  | Fungi | | |
| --- | --- | --- | --- | --- | --- | --- | --- |
|  | Rhizosphere | Endosphere | *P* Value |  | Rhizosphere | Endosphere | *P* Value |
| OTUs | 2015.48 ± 176.58 | 1728.34 ± 214.67 | 0.006** |  | 1027.58 ± 98.79 | 934.75 ± 107.26 | 0.045* |
| Chao | 1156.01 ± 166.18 | 988.31 ± 103.92 | 0.037* |  | 620.06 ±85.50 | 482.33 ±77.22 | 0.002** |
| Shannon | 5.21 ± 0.75 | 3.98 ± 0.76 | 0.021* |  | 3.63 ±0.64 | 2.76 ± 0.51 | 0.036* |

Data represents as mean ± Standard Deviation (S.D.). Statistical analyses were performed with Student's t-test between the two groups. The number of OTUs, richness estimator Chao, and community diversity estimator Shannon were calculated. n = 9, in each group. **P* < 0.05, ***P* < 0.01 and ****P* < 0.001 rhizosphere vs. endosphere group.

**Table S2.** Topological properties of the empirical ecological networks (MENs) of root-associated microbiomes and their associate random MENs.

| Networks | No. of original genera^a^ | Network size (n)^b^ | Total links | Similarity threshold (St) | R^2^ of Power law | Avg path distance (GD) | Avg cluster coefficient (avgCC) | Modularity (M) |
| --- | --- | --- | --- | --- | --- | --- | --- | --- |
| Empirical networks | 200 | 135 | 265 | 0.78 | 0.821 | 6.391 | 0.291 | 0.649 |
| Random networks | 200 | -^c^ | -^c^ | -^c^ | -^c^ | 3.460 ± 0.087 | 0.064 ± 0.013 | 0.462 ± 0.009 |

^a^The number of genera that were originally used for network construction using the random matrix theory (RMT)-based approach.

^b^The number of genera (i.e., nodes) in the network.

^c^The parameters are not applicable.

**

**

**Fig. S1.** The rarefaction curve analysis of the bacterial (a) and fungal (b) sequences. The curves were constructed using the Shannon index values of the OTUs and the number of reads.

**
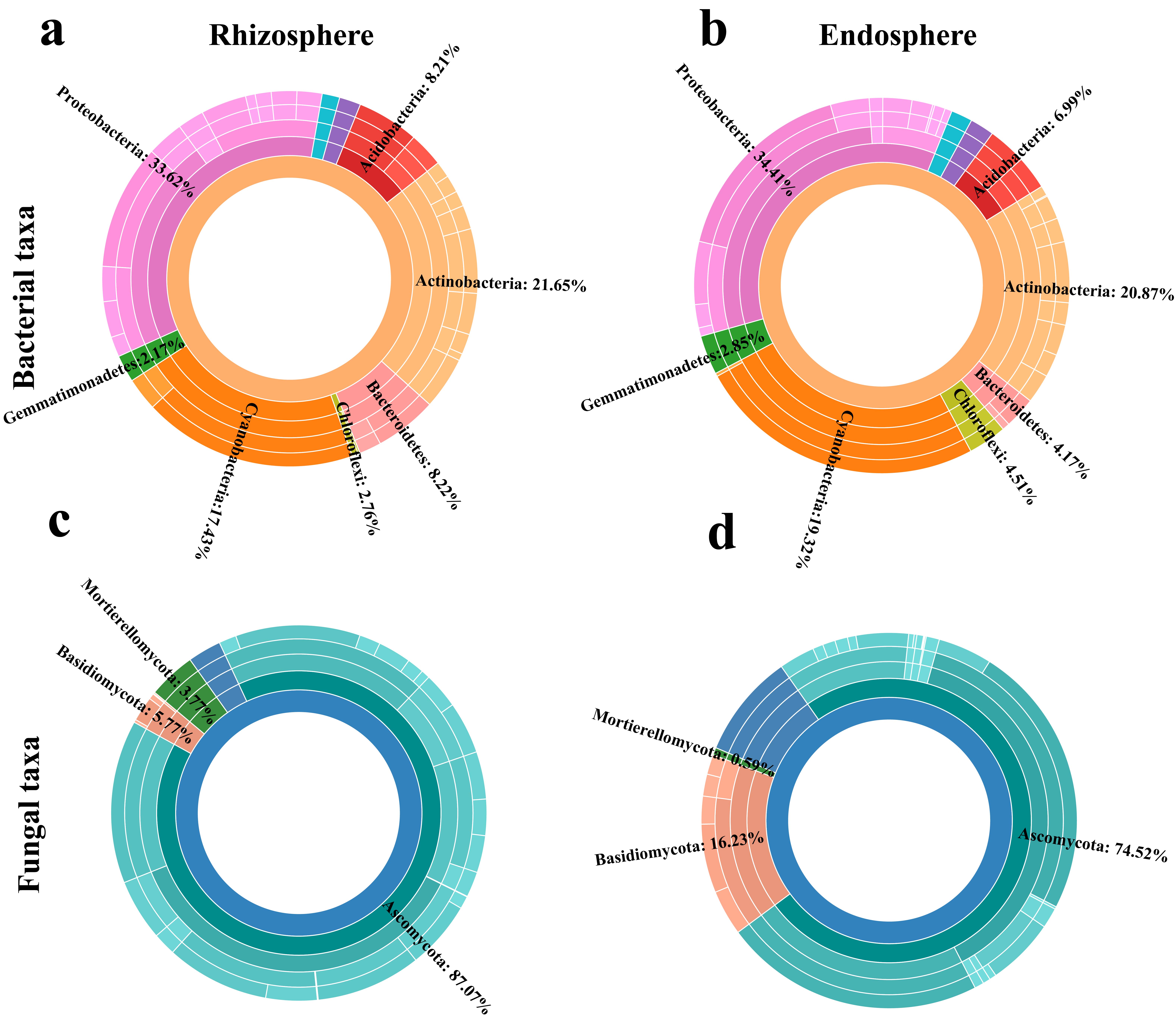
**

**Fig. S2.** Sunburst showing the hierarchical taxonomic composition of bacteria (a, b) and fungi (c, d). From the inner circle to the outer circle, the phylogenetic levels of kingdom, phylum, class, order, and family are displayed in order. The colors were assigned automatically to distinguish taxa. a, c, The hierarchical taxonomic composition of rhizosphere samples. b, d, The hierarchical taxonomic composition of endosphere samples.


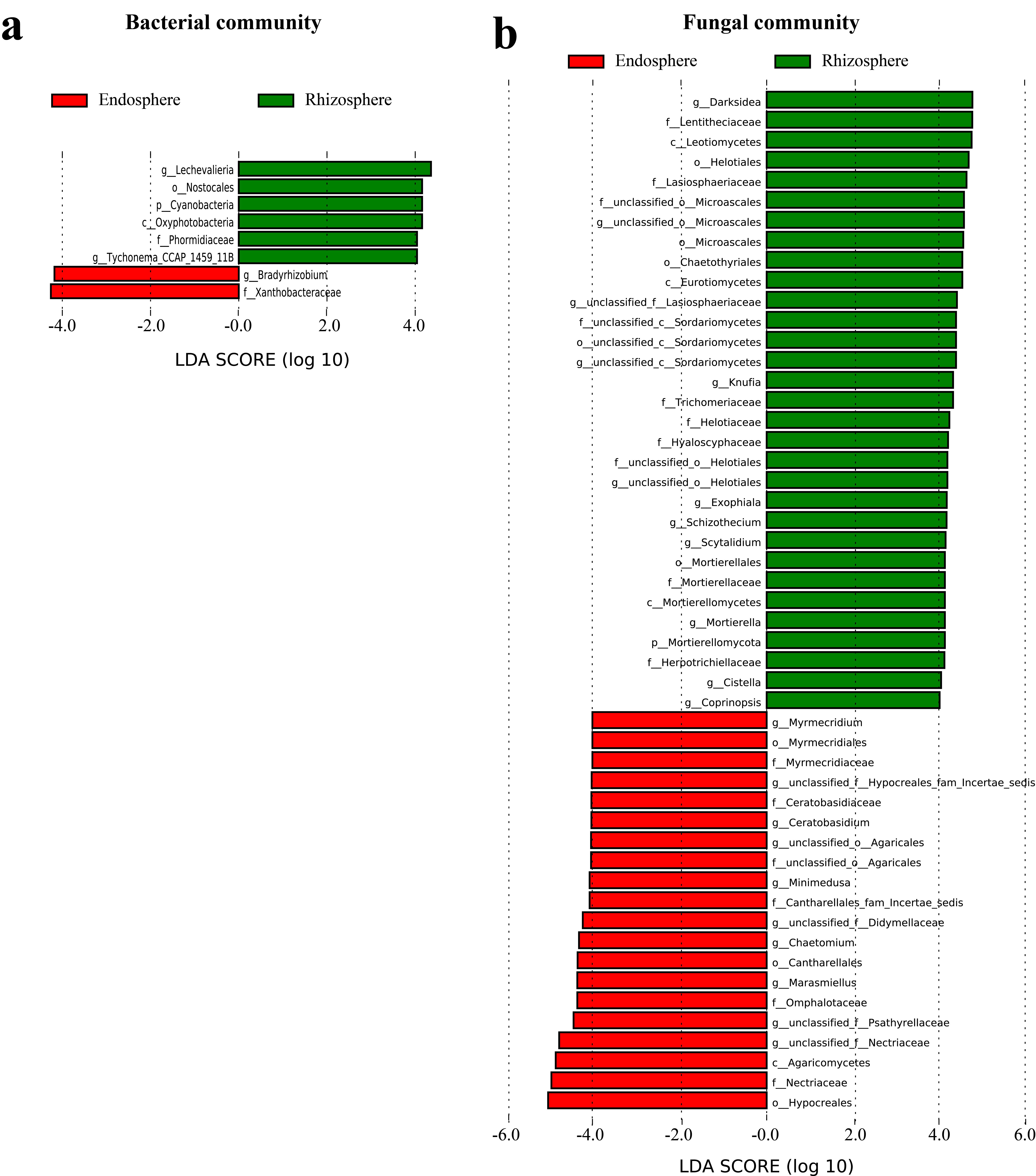


**Fig. S3.** Enriched taxa of the bacterial (a) and fungal (b) communities reaching a linear discriminant analysis (LDA) significance threshold of 4.0.
